# CHIR99021 enhances *Klf4* Expression through *β*-Catenin Signaling and miR-7a Regulation in J1 Mouse Embryonic Stem Cells

**DOI:** 10.1371/journal.pone.0150936

**Published:** 2016-03-03

**Authors:** Zhiying Ai, Jingjing Shao, Yongyan Wu, Mengying Yu, Juan Du, Xiaoyan Shi, Xinglong Shi, Yong Zhang, Zekun Guo

**Affiliations:** 1 College of Life Sciences, Northwest A&F University, Yangling, Shaanxi, China; 2 College of Veterinary Medicine, Northwest A& F University, Yangling, Shaanxi, China; 3 Key Laboratory of Animal Biotechnology, Ministry of Agriculture, Northwest A& F University, Yangling, Shaanxi, China; University of Kentucky, UNITED STATES

## Abstract

Understanding the mechanisms that regulate pluripotency of embryonic stem cells (ESCs) is important to ensure their safe clinical use. CHIR99021 (CHIR)-induced activation of Wnt/*β*-catenin signaling promotes self-renewal in mouse ESCs (mESCs). *β*-catenin functions individually or cooperates with transcription factors to activate stemness factors such as c-Myc, Esrrb, Pou5f1, and Nanog. However the relationship between the core pluripotent factor, Kruppel-like factor 4 (also known as GKLF or EZF) and Wnt/*β*-catenin signaling, remains ambiguous in J1 mESCs. DNA microarray analysis revealed that CHIR-treatment promoted pluripotency-maintaining transcription factors and repressed germ layer specification markers. CHIR also promoted genes related to the development of extracellular regions and the plasma membrane to maintain pluripotency of J1 mESCs. Among the CHIR-regulated genes, *Klf4* has not been reported previously. We identified a novel cis element in the *Klf4* gene that was activated by *β*-catenin in J1 mESCs. We determined that *β*-catenin interacted with this cis element, identifying *Klf4* as a *β*-catenin target gene in this context. Moreover, several microRNAs that targeted the 3′-UTR of *Klf4* mRNA were identified, with miR-7a being down-regulated by CHIR in a *β*-catenin-independent manner in J1 mESCs. These data collectively suggest that CHIR enhances *Klf4* expression by repressing miR-7a expression or canonical Wnt pathway activation.

## Introduction

Embryonic Stem Cells (ESCs) are pluripotent cells derived from the inner cell mass of mammalian embryos, and can indefinitely expand in cultures through symmetrical self-renewal divisions [1, 2]. The self-renewability of mouse ESCs (mESCs) can be maintained in serum-containing medium supplemented with leukemia inhibitory factor (LIF) or serum-free N2B27 medium in the presence of two small molecule inhibitors (2i), CHIR and PD0325901 (PD) [1, 3]. LIF, in conjunction with bone morphogenetic protein (BMP)-4 or fetal bovine serum, primarily acts through the JAK-STAT3 and BMP-SMAD signaling pathways to maintain the self-renewal of mESCs [3]. This potential self-renewability is further enhanced by combined use of CHIR and PD, two inhibitors that inhibit glycogen synthase kinase-3 (GSK3) and mitogen-activated protein kinase (ERK1/2) signaling, respectively [4]. The inhibition of GSK3 regulates canonical Wnt/*β*-catenin signaling to stimulate self-renewal of ESCs through stabilization of *β*-catenin [5]. The stabilized *β*-catenin by GSK3 inhibition enters into the nucleus and functions individually or interacts with transcription factors to reinforce pluripotency by activating stemness factors such as *c*-Myc, Esrrb, Oct4 (also known as Pou5f1), and Nanog [6, 7]. Recently, it has been demonstrated that *Klf4* mRNA can be promoted by CHIR treatment in B6 mESCs [8, 9]. Thus, there likely exists a potential molecular regulation mechanism between CHIR and *Klf4*.

*Klf4*, a member of the Kruppel-like factor (Klf) family of conserved zinc finger transcription factors, establishes an ‘‘authentic” and ‘‘metastable” pluripotent state in various pluripotent cell types [10–12]. *Klf4* also mediates the basic nuclear organization at the *Oct4* locus and maintains a high-order chromatin structure, which contributes to maintaining the pluripotency of ESCs [13]. As a direct downstream target of LIF signaling, *Klf4* is indispensable to maintaining the self-renewability and pluripotency of mESCs [14]. In serum-free culture in the presence of 2i (N2B27+PD+CHIR), ESCs can be maintained in a self-renewal state, even if CHIR is replaced by LIF (N2B27+ PD+LIF) [15]. These results imply that CHIR may act on *Klf4* and take over the functions of LIF in mESCs.

MicroRNAs (miRNAs) are evolutionarily conserved, small noncoding RNAs consisting of 21–25 nucleotides, that have essential roles in the self-renewability of ESCs [16–19]. For example, the loss of DiGeorge syndrome critical region gene 8 (Dgcr8), which is required for miRNA biogenesis, results in an inability to silence the self-renewal program of ESCs when they are placed in differentiation-inducing conditions [20]. Moreover, the introduction of certain miRNAs can target the ESC transcriptional network and regulate the self-renewability of ESCs [21, 22]. Interestingly, ESC transcription factors are typically associated with promoters of miRNAs that are preferentially expressed in ESCs [23]. These data suggest that miRNAs can integrate into the regulatory circuitry-controlling self-renewability of ESCs. In this study, we identified transcription factor *Klf4* as a downstream target of CHIR, whose expression and functions are regulated by miR-7a and the Wnt/*β*-catenin signaling pathway.

## Results

### Identification of genes differentially induced/suppressed in J1 mESCs by CHIR

**Fig 1:**
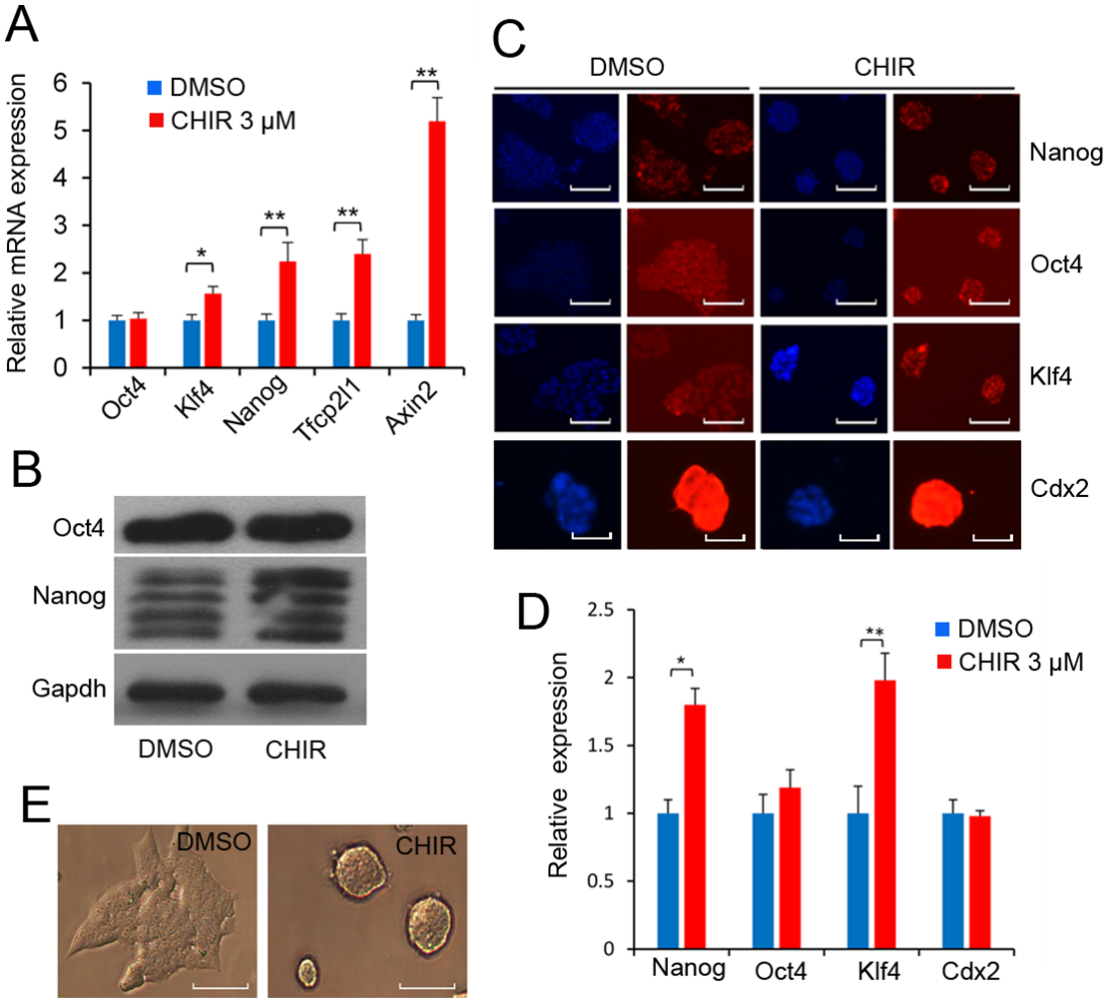
CHIR99021 combined with LIF maintains J1 mESC pluripotency under feeder-free conditions. **(A)**: CHIR regulates pluripotent marker in J1 mESCs. J1 mESCs were treated with the indicated concentration of CHIR for 24 h. qPCR validation of *Oct4*, *Nanog*, *Klf4*, *Tfcp2l1* and *Axin2* using the comparative Ct method. Data are presented as the mean ± SD of three independent experiments (*p < 0.05; **p < 0.01). Gapdh was used to normalize template levels. **(B)**: Western blot analysis of Oct4 and Nanog in J1 mESCs in the presence of 1,000 U/ml LIF and with or without 3 μM CHIR for 24 h. **(C)**: Immunofluorescence staining of pluripotent markers. J1 mESCs were treated with or without 3 μM CHIR for 24 h, and pluripotent markers Oct4, Klf4, Nanog and Cdx2 were analyzed by immunofluorescence staining. Nuclei were stained with DAPI. Scale bars represent 50 μm. **(D)**: Quantification of Oct4, Klf4, Nanog and Cdx2 signal intensities in DMSO- and CHIR-treated J1 mESCs by ImageJ software. Labeling intensity was expressed relative to that of the DMSO-treated mESCs (set as 1). The experiments were replicated 3 times. In each replication, n = 100–150 per group. *p < 0.05. **(E)**: CHIR promoted compact colony morphology of J1 mESCs. J1 mESCs were treated with 3 μM CHIR or DMSO for 24 h, and cell morphology was detected under phase contrast microscopy. Scale bars represent 50 μm.

**Fig 2:**
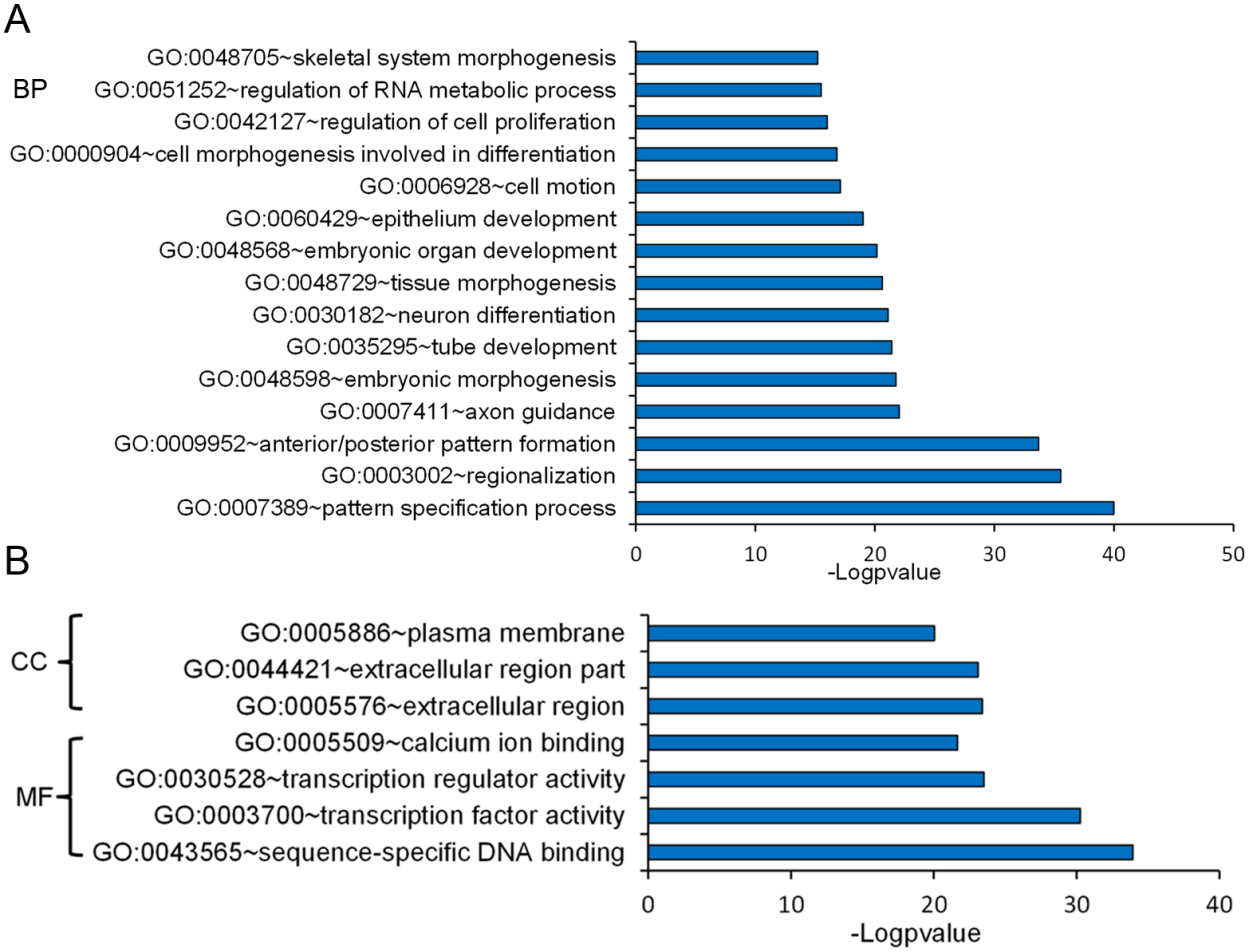
GO classification of differentially expressed genes. **(A)**: GO classification of the biological processes (BP). The GO term (FDR < 0.05) comparison of differently expressed genes (fold change > 2, p < 0.05) identified by gene expression microarray. Data are presented as a histogram of the relevant BP identified and shown as the−log ^p-value^. **(B)**: GO classification of the molecular function (MF) and cellular component (CC). GO classification of the MF and CC genes (fold change > 2, p < 0.05) using DAVID. Data are presented in a histogram of the relevant identified CC and MF, shown as the −log^p-value^.

We compared the expression profiles of J1 mESCs (GEO ID Number: GSE40959) treated with dimethyl sulphoxide (DMSO) or 3 μM CHIR. CHIR can promote the expression of pluripotent factors such as *Nanog*, *Klf4*, *Tbx3*, *Tfcp2l1*, *Nr5a2*, *Nr0b1*, and *Esrrb*, and repress germ layer specification markers such as *Gata3*, *Nodal*, *Otx2*, *Pax6*, *Notch1*, and *Neurod1* [9]. The CHIR-induced upregulation of pluripotent markers was confirmed by quantitative real-time polymerase chain reaction (RT-qPCR) analysis, western blot analysis and immunofluorescence staining in J1 mESCs. We found that 3 μM CHIR treatment elevated the expression levels of Nanog, Klf4 and *Tfcp2l1* (Fig 1A, 1B and 1C), while trophectoderm marker Cdx2 expression appeared to be unchanged (Fig 1C). However, *Oct4* mRNA expression level was not influenced by CHIR treatment (Fig 1A). Meanwhile, 3 μM CHIR treatment enhanced compact colony morphology, which became smooth and tightly protuberant after the addition of CHIR (Fig 1E). We then focused on differentially expressed genes with five-fold or higher fold changes in the microarray data and identified 74 up-regulated genes and 39 down-regulated genes, following 3 μM CHIR treatment (S1 Table). Among these differentially expressed genes, the most highly expressed transcription factor, Tfcp2l1, is reported to be directly linked to the pluripotent factor Nanog [24]. To avoid a biased interpretation, we performed gene ontology (GO) annotation analysis of differentially expressed genes. Biological process analysis revealed that differentially expressed genes principally participated in cell proliferation, neuron differentiation and RNA metabolism (Fig 2A). Moreover, GO analysis showed that the CHIR-modulated genes were mainly enriched in the extracellular regions and plasma membrane (Fig 2B). This characteristic distribution of cellular components may be the reason for the change of colony morphology of J1 mESCs after CHIR treatment [25]. These data demonstrate that CHIR reinforces ESC pluripotency by regulating the expression of stemness factors such as Klf4, thus maintaining colony morphology and promoting ESC propagation.

### CHIR enhances *Klf4* expression in J1 mESCs

**Fig 3:**
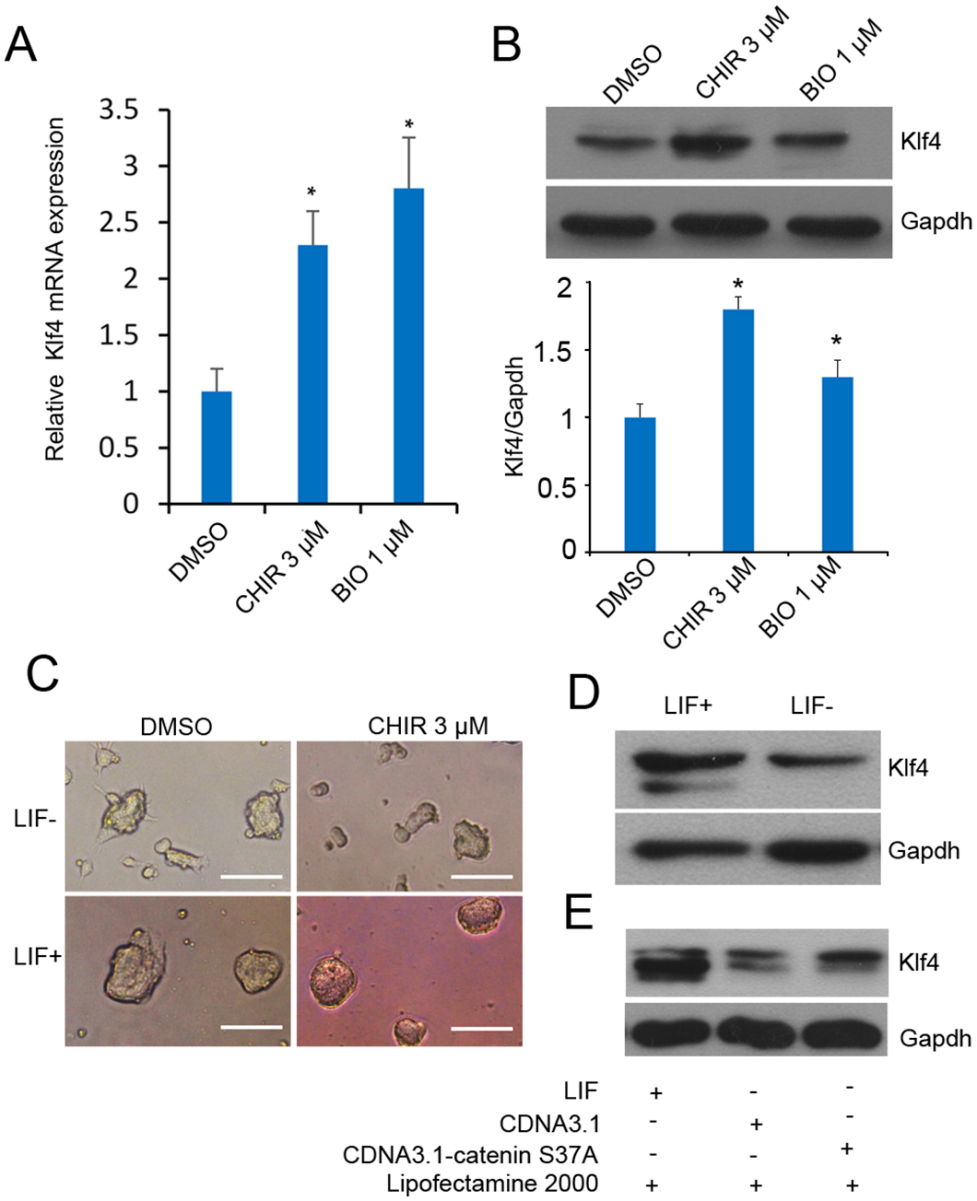
CHIR influences *Klf4* expression in ESCs. **(A)**: CHIR promotes *Klf4* mRNA expression. J1 mESCs were cultured in LIF-containing medium with or without 3 μM CHIR for 24 h, and *Klf4* expression level was analyzed by RT-qPCR using the comparative Ct method. *Gapdh* was used to normalize template levels. Data are presented as the mean ± SD of three independent experiments (*p < 0.05; **p < 0.01). Small molecule BIO (1 μM) was used as a control. **(B)**: CHIR promotes Klf4 expression. Representative western blot analysis of Klf4 in J1 mESCs in the presence of 1,000 U/ml LIF, with or without 3 μM CHIR. Small molecule BIO (1 μM) was used as control. Cell lysates were extracted and analyzed by western blot, the experiments were repeated for three times. The graph presents Klf4 levels normalized to corresponding Gapdh levels, error bar indicates standard deviation (*p < 0.05). **(C)**: CHIR partly rescued the colony morphology changes without LIF. J1 mESCs were treated with 3 μM CHIR or DMSO with or without LIF for 24 h, cell morphology was detected under phase contrast microscopy. Scale bars represent 50 μm. **(D)**: The expression of Klf4 is downregulated without LIF. Western blot analysis of Klf4 in J1 mESCs in the presence or absence of 1,000 U/ml LIF for 24 h. **(E)**: *β*-catenin elevates Klf4 expression in the absence of LIF. J1 mESCs were transfected with *β*-catenin expression vector pCDNA3.1-*β*-catenin s37a and the negative control pCDNA3.1 in the presence or absence of LIF. At 48 h of incubation, Klf4 expression level was analyzed by western blot. Gapdh was used to normalize template levels.

The microarray data showed that CHIR positively regulated *Klf4* mRNA expression. The differential expression level of *Klf4* was confirmed by RT-qPCR in J1 mESCs. As shown in Fig 3A, levels of endogenous *Klf4* mRNA were elevated 2-fold compared with control DMSO-treated cells. The changes in *Klf4* mRNA expression correlated with altered Klf4 protein abundance after 3 μM CHIR treatment in mESCs (Fig 3B). 6-Bromo-indirubin-3′-oxime (BIO), an inhibitor of GSK3, was used as a positive control [26] (Fig 3A and 3B). To determine whether CHIR could promote the expression of *Klf4* in differentiated mESCs, we first confirmed that without LIF for 24 h, mESCs showed differentiation characteristics in the colony morphology of J1 mESCs (Fig 3C). Meanwhile, Klf4 expression was repressed without LIF (Fig 3D). However, CHIR elevated the expression of *Klf4* in the absence of LIF (Fig 3E and S1 Fig).

**Fig 4:**
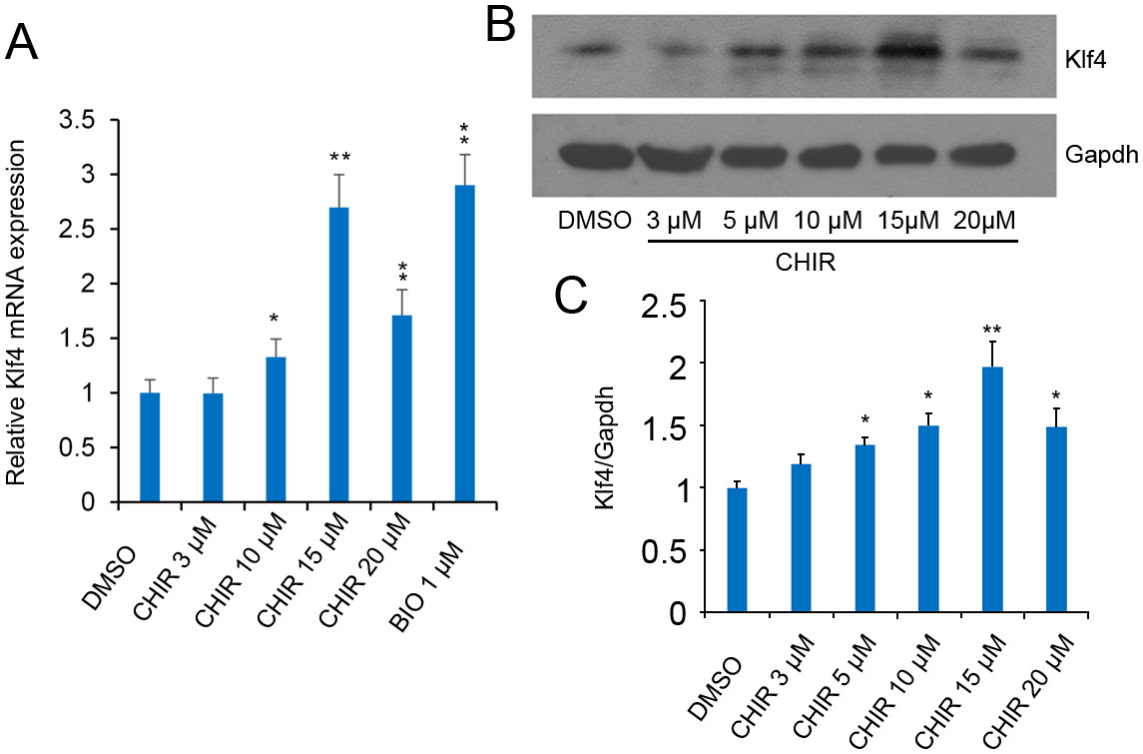
CHIR influences *Klf4* expression in F9 EC cells. **(A)**: CHIR promotes *Klf4* mRNA expression. F9 EC cells were treated with CHIR at a final concentration of 3, 5, 10, 15 and 20 μM respectively for 24 h, and *Klf4* expression level was analyzed by RT-qPCR using the comparative Ct method. Gapdh was used to normalize template levels. Data are presented as the mean ± SD of three independent experiments (*p < 0.05; **p < 0.01). **(B)**: CHIR promotes Klf4 expression. Representative western blot analysis of Klf4 in F9 EC cells after treatment with or without different indicated concentrations of CHIR for 48 h. Cell lysates were extracted and analyzed by western blot. Relative expression levels were compared with Gapdh, the experiments were repeated for three times. **(C)**: Quantification of Klf4 in CHIR-treated J1 mESCs. The graph presents Klf4 levels normalized to corresponding Gapdh levels. Labeling intensity was expressed relative to that of the DMSO-treated mESCs (set as 1), error bar indicates standard deviation (*p < 0.05; **p < 0.01).

To verify that the effects were not constrained to mESCs, we used mouse F9 embryonal carcinoma (EC) cells for further detection. F9 EC cells were treated with CHIR at final concentrations of 3, 10, 15 and 20 μM respectively. After 24 h treatment of F9 EC cells, levels of endogenous *Klf4* were elevated at different concentrations of CHIR. *Klf4* mRNA (Fig 4A) and protein (Fig 4B) expression levels peaked in the presence of 15 μM CHIR treatment. Collectively, these results suggest that CHIR treatment positively regulates expression of endogenous Klf4.

### CHIR Regulates *Klf4* expression by canonical Wnt pathway activation

As indicated above, Klf4 expression was responsive to CHIR and BIO-activated signaling. We therefore explored the mechanism by which CHIR influences *Klf4* expression in J1 mESCs. We considered *β*-catenin as the key regulator, because this protein works as a dominant downstream transcription factor of Gsk3*β*. Previous studies have demonstrated that *β*-catenin activates canonical Wnt/*β*-catenin signaling by binding to the promoter of the gene of interest in a T-cell factor (Tcf)/lymphoid enhancer factor (Lef)-dependent manner [27, 28].

**Fig 5:**
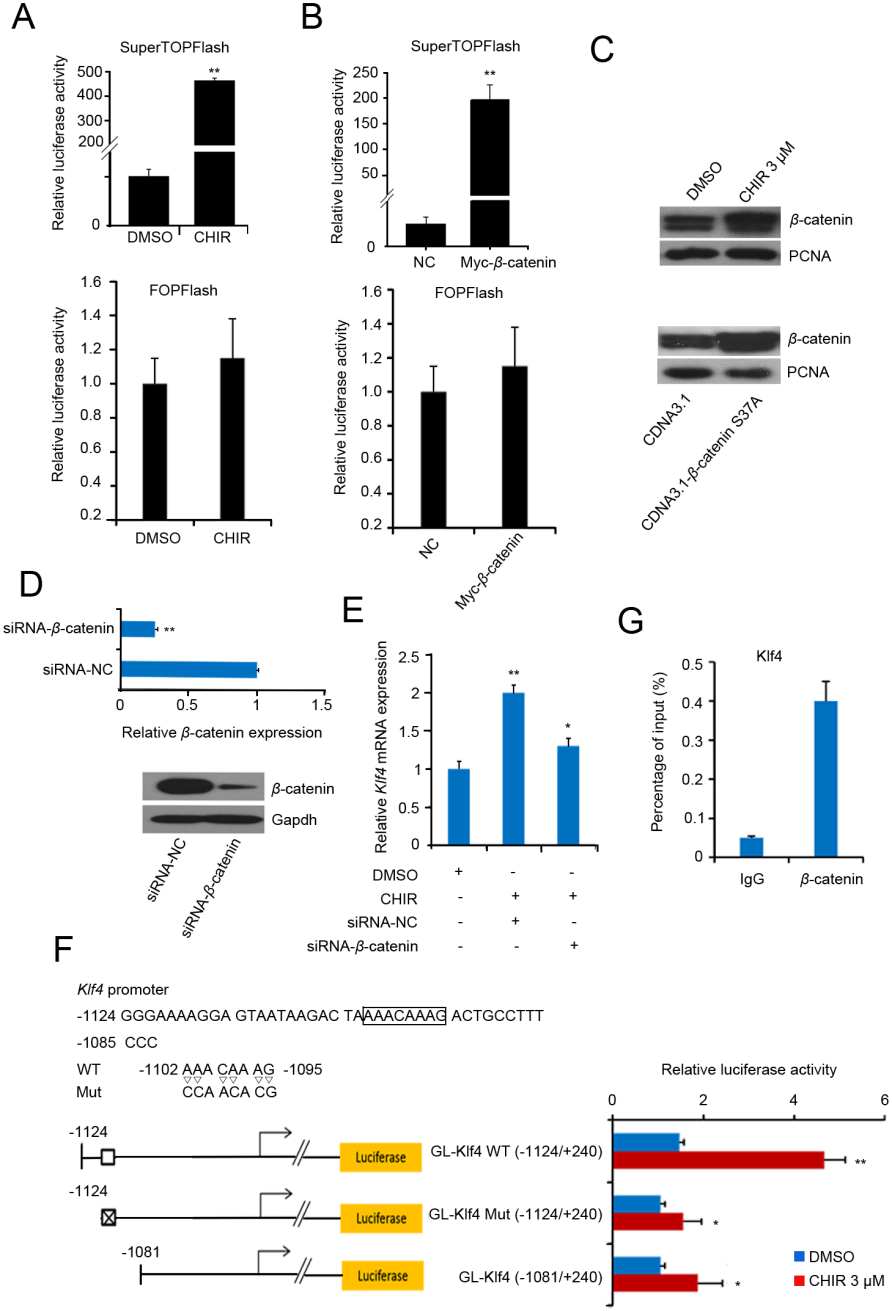
CHIR regulates *Klf4* expression by canonical Wnt pathway activation. **(A)**: TopFlash and FopFlash assay after CHIR treatment. J1 mESCs were transfected with pSuperTOPFlash reporter plasmids or the pTA-luc control plasmid. At 5 h after transfection, fresh medium was added and 3 μM CHIR or an equal volume of DMSO was added to the transfected cells. 24 h after transfection, luciferase activity was detected using the dual-luciferase reporter assay. **(B)**: TopFlash and FopFlash assay after overexpression of *β*-catenin. pCMV-Myc or pCMV-Myc-*β*-catenin was co-transfected with pSuperTOPFlash reporter plasmids or pTA-luc control plasmid into J1 mESCs, followed by 24 h of incubation. Luciferase activity is expressed relative to that of pTA-luc. Data are presented as the mean ± SD of three independent experiments. **(C)**: CHIR treatment or *β*-catenin overexpression promotes nuclear *β*-catenin expression. J1 mESCs were treated with 3 μM CHIR or equal volume of DMSO (upper panel), or transfected with pCDNA3.1-*β*-catenin s37a / pCDNA3.1 control plasmid (down panel) for 48 h, The nucleus protein were extracted and the expression of *β*-catenin was analyzed by western blot. Relative expression levels were compared with PCNA. **(D)**: *β*-catenin knockdown. Cells were transfected with siRNA-*β*-catenin or NC for 48 h, and RT-qPCR (upper panel) or western blot (lower panel) was used to detect the knockdown efficiency of *β*-catenin. **(E)**: Knockdown of *β*-catenin represses *Klf4* expression. J1 mESCs were transfected with siRNA-*β*-catenin or siRNA-NC. At 5 h after transfection, fresh medium was added and 3 μM CHIR or an equal volume of DMSO was added to the transfected cells, followed by 48 h of incubation. *Klf4* expression was validated by qPCR. Gapdh was used to normalize template levels. **(F)**: A novel cis element in the *Klf4* gene was activated by *β*-catenin. The cis-element (WT) and its mutational type (Mut) are shown in the upper panel. A schematic representation of the promoter structure of *Klf4* is shown in the lower panel. Control plasmid pGL4.10 was co-transfected with pGL-*Klf4* (−1124/+240) or pGL-*Klf4* (−1081/+240) promoter reporter plasmid into J1 mESCs. At 5 h after transfection, fresh medium was changed and 3 μM CHIR or an equal volume of DMSO was added to the transfected cells, followed by 24 h of incubation. Luciferase activity is expressed relative to that of pGL4.10. Data are presented as the mean ± SD of three independent experiments. **(G)**: Chromatin immunoprecipitation assay for the detection of cis-element in *Klf4* gene. ChIP was performed using anti-β-catenin antibody and anti-IgG as a control antibody to detect enriched fragments. Data are presented as the mean ± SD of three independent experiments. (*p < 0.05; **p < 0.01).

To detect whether *Klf4* expression is induced by the activated canonical Wnt signaling, we used a construct with seven tandem copies of the consensus Tcf/Lef binding site (TopFlash) (S2 Fig) for the Tcf-responsive TOPFlash reporter assay. J1 mESCs were co-transfected with TopFlash and a gene construct expressing *β*-catenin, Fopflash was used as a negative control. We first confirmed that CHIR treatment or *β*-catenin overexpression activated the canonical Wnt/*β*-catenin signaling using the TOPFlash reporter assay (Fig 5A and 5B). Furthermore, CHIR treatment or *β*-catenin overexpression led to the accumulation of cytosolic *β*-catenin (S3 Fig), which translocated into the nucleus (Fig 5C) and formed the *β*-catenin/Tcf/Lef complex to activate Wnt targets. We then examined whether the activation of Klf4 (Fig 1A and 1C) mediated by CHIR required the *β*-catenin-dependent signaling pathway. We therefore attempted to deplete *β*-catenin by transfecting siRNA specific for *β*-catenin (siRNA-*β*-catenin) into J1 mESCs. RT-qPCR analysis revealed that the depletion of *β*-catenin was significant (Fig 5D, upper panel). Western blot with a *β*-catenin-specific antibody demonstrated that the endogenous *β*-catenin protein was significantly depleted in siRNA-*β*-catenin-transfected ESCs (Fig 5D, lower panel). We then investigated whether *Klf4* expression was reduced after *β*-catenin knockdown in CHIR-treated mESCs. We found that *β*-catenin knockdown reduced *Klf4* expression even in CHIR-treated ESCs (Fig 5E). These results indicate that CHIR promotes *Klf4* by stabilizing *β*-catenin and activating canonical Wnt/*β*-catenin signaling.

We then screened the mouse *Klf4* 5′ flanking region for sequences corresponding to the *β*-catenin-Tcf/Lef DNA-binding consensus sequences to determine the mechanism by which *β*-catenin increased *Klf4* expression in J1 mESCs. We noted a potential Tcf/Lef binding site (−1102/−1095) in the *Klf4* promoter according to the prediction results by PROMO v8.3 [29, 30]. We cloned the promoter fragment (−1124/+240) containing the Tcf/Lef binding site (wild type or mutant type) and the truncated promoter (−1081/+240) into the pGL4.10 luciferase reporter vector to confirm the binding site through dual luciferase reporter assays. As shown in Fig 5F, the *Klf4* promoter (−1124/+240) responded to the CHIR signaling depending on the Tcf/Lef binding sites. However, the truncated promoter (−1081/+240) and the *Klf4* promoter-mut (−1124/+240) were slightly elevated after CHIR treatment, this phenomenon cannot disregard the indirect regulatory role on *Klf4* by other factors that respond to CHIR signaling. To further characterize the DNA-binding sequence that is recognized by *β*-catenin, we performed chromatin immunoprecipitation assays. As shown in Fig 5G, a 7.6-fold enrichment of *β*-catenin was observed. These results collectively indicate that *β*-catenin directly binds to the *Klf4* promoter and activates the expression of *Klf4*.

### CHIR regulates Klf4 expression by repressing miRNA expression

**Fig 6:**
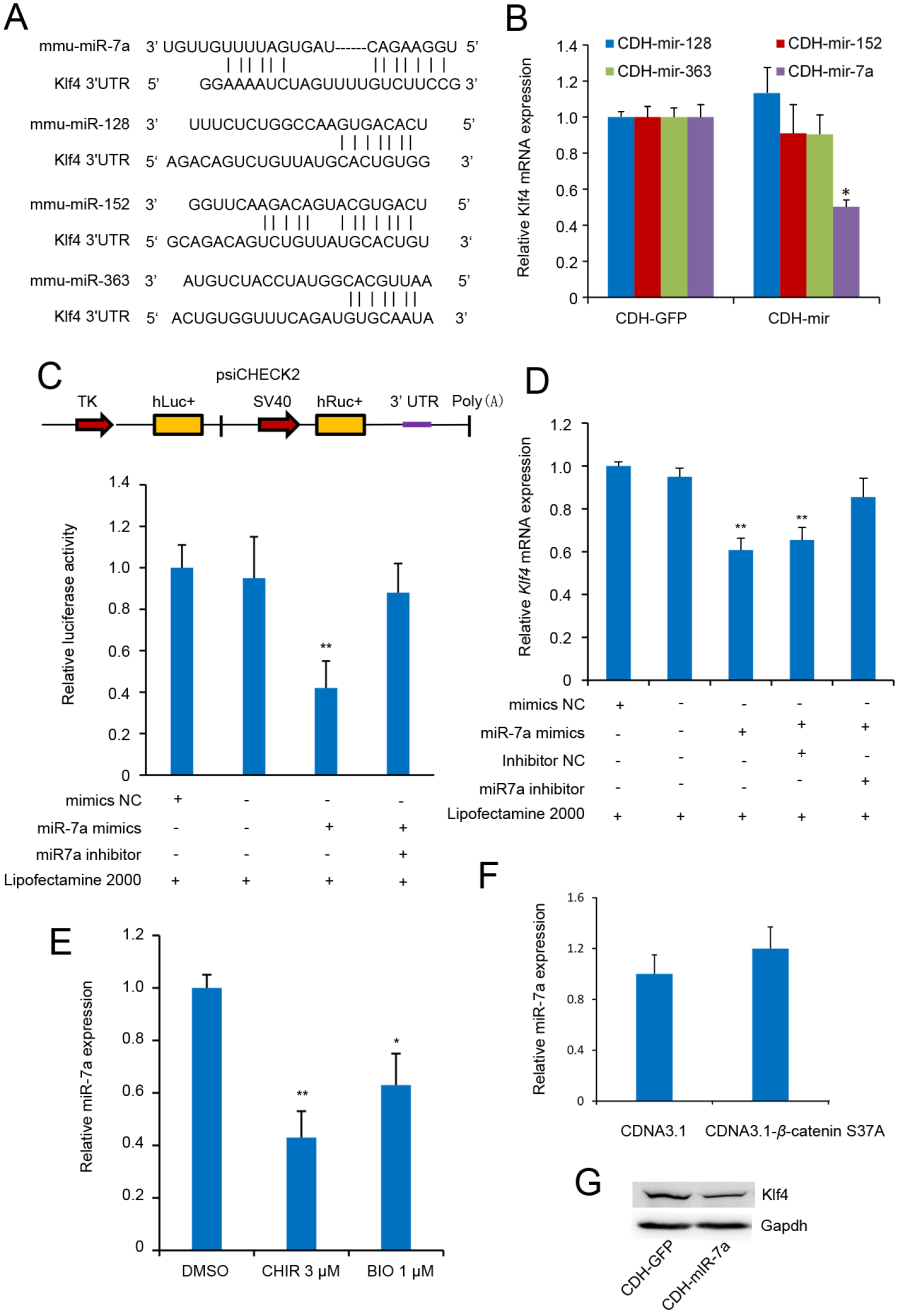
Klf4 is directly targeted by miR-7a. **(A)**: Schematic of miRNA binding sites in the *Klf4* gene. Presentation of miR-7a, miR-125, miR-152, and miR-363 target sites in the 3′-UTR of *Klf4*. The lines represent sequence alignment of predicted miRNA binding sites on *Klf4* 3′-UTR. **(B)**: miRNAs regulate *Klf4* expression. J1 mESCs were transfected with pCDH-CMV-pre-miRNA-EF1-coGFP or pCDH-CMV-MCS-EF1-coGFP control plasmid. After 24 h of incubation, *Klf4* expression level was analyzed by RT-qPCR. Gapdh was used to normalize template levels. **(C)**: miR-7a mimics regulate *Klf4* expression in a post-transcriptional regulation manner. Schematic representation of the 3′-UTR reporter constructs in the upper panel. Abbreviations TK, hluc+, SV40 and hRluc represent HSV-TK promoter, firefly luciferase gene, SV40 early enhancer/promoter, and Renilla luciferase gene, respectively. In the lower panel, psiCHECK2-*Klf4*-3′UTR or psiCHECK2 control plasmids were co-transfected with mimics NC or miR-7a mimics/inhibitor into 293FT cells. At 36 h after incubation, luciferase activity was expressed relative to that of psiCHECK2. **(D)**: miR-7a mimics regulates *Klf4* expression. J1 mESCs were transfected with mimics NC or miR-7a mimics/inhibitor. At 36 h of incubation, *Klf4* expression level was analyzed by RT-qPCR. Gapdh was used to normalize template levels. **(E)**: CHIR and BIO inhibit miR-7a expression. J1 mESCs were cultured in LIF-containing medium with or without CHIR/BIO for 24 h, and miR-7a expression level was analyzed by RT-qPCR. U6 was used to normalize template levels. Data are presented as the mean ± SD of three independent experiments (*p < 0.05; **p < 0.01). **(F)**: *β*-catenin does not influence miR-7a expression. J1 mESCs were transfected with *β*-catenin expression vector pCDNA3.1-*β*-catenin s37a and the negative control pCDNA3.1. At 48 h of incubation, miR-7a expression level was analyzed by RT-qPCR. Gapdh was used to normalize template levels. Data are presented as the mean ± SD of three independent experiments. **(G)**: miR-7a represses *Klf4* expression. J1 mESCs were transfected with miR-7a expression vector pCDH-mir-7a and the negative control pCDH-GFP. At 48 h of incubation, *Klf4* expression level was analyzed by western blot. Gapdh was used to normalize template levels.

To further decipher the possible role of CHIR in *Klf4* expression, we investigated the effects of miRNAs on the regulation of *Klf4*. miRNAs that potentially targeted the 3′-UTR of *Klf4* were predicted using multiple databases including TargetScan, Pic Tar and miRanda (S2 Table). Four candidates predicted by at least two databases each were selected for further investigation (Fig 6A). To test whether the predicated miRNAs were functional, we transfected the lentivirus expression vectors carrying the candidate miRNAs into J1 mESCs. We found that only miR-7a significantly suppressed *Klf4* expression (Fig 6B and S4 Fig). However, the other three miRNAs had no inhibitory effect on *Klf4*. These results demonstrated that miR-7a may be a potential miRNA that represses the expression of *Klf4*; therefore, we selected miR-7a for further investigation. To further confirm that miR-7a was functional for *Klf4*, we subcloned the 3′-UTR fragment of *Klf4* downstream the reporter gene into the psiCHECK-2 vector (Fig 6C, upper panel). The reporter vector contained the full-length mouse *Klf4* 3′-UTR sequence, which was cloned downstream of the reporter gene Renilla, so that reporter gene expression was regulated by the *Klf4* 3′-UTR sequence. Luciferase assays were performed by co-transfecting the reporter vector and miR-7a mimics into 293FT cells. As shown in Fig 6C, the reporter harboring the 3′-UTR fragment of *Klf4* was significantly repressed, whereas miR-7a inhibitor rescued luciferase activity. Furthermore, transfection of miR-7a mimics suppressed *Klf4* mRNA levels in J1 mESCs, as analyzed by RT-qPCR. Consistently, *Klf4* upregulation was detected when miR-7a was blocked in J1 mESCs using specific antisense inhibitors (Fig 6D). Thus, these results indicate together with previous reports [31, 32] that miR-7a represses *Klf4* expression. Because *Klf4* expression was promoted by CHIR, we asked whether CHIR enhanced *Klf4* expression by repressing the level of miR-7a that targeted *Klf4*. We performed an RT-qPCR assay and found that CHIR inhibited miR-7a expression (Fig 6E), while BIO was used as a positive control. However, overexpression of β-catenin was unable to repress miR-7a expression (Fig 6F). Thus, these results suggest that CHIR regulates *Klf4* expression mediated by miR-7a in a *β*-catenin-independent manner.

## Discussion

CHIR is a potent agonist of the Wnt signaling pathway by inhibiting GSK3, which phosphorylates and degrades *β*-catenin. The accumulation of cytoplasmic *β*-catenin enables its nuclear entry and cooperates with Tcf/Lef for the maintenance of pluripotency-associated genes such as s *c*-Myc, *Esrrb*, *Oct4*, and *Nanog* in mESCs [33, 34]. However, CHIR induces differentiation in human ESCs [35]. This result may be partly attributable to the fact that human ESCs more closely resemble mouse epiblast stem cells that correspond to a slightly later developmental stage than those in the inner cell mass. In this study, we demonstrated that CHIR was able to reinforce J1 mESC pluripotency, not only by promoting the pluripotent network, but also by strengthening propagation of mESCs and consolidating biosynthetic capacity (Figs 1 and 2), consistent with previous observations [4]. We also found that CHIR was able to promote *Klf4* expression (Fig 1A), consistent with previous studies in which *Klf4* was found to be promoted by CHIR in B6 ESCs [8]. Thus, it is likely that a potential molecular regulation mechanism might exist between CHIR and *Klf*4. Meanwhile, *Klf4* is reported to be a downstream effector of LIF signaling, which is indispensable to maintain ESC pluripotency [36]. Moreover LIF can substitute CHIR under serum-free N2B27/2i conditions [24]. These results imply that LIF and CHIR could have the same effects on *Klf4* expression, further suggesting that a relationship might exist between *Klf4* and CHIR.

In this study, we showed that CHIR enhanced *Klf4* expression through *β*-catenin signaling and miR-7a regulation in J1 mESCs. Moreover, CHIR treatment could lead to the enhanced compact colony morphology in a LIF-dependent manner, implying a collaborative effect between CHIR and LIF/STAT3 signaling in mESCs (Fig 3C). Interestingly, *Klf4*, as a missing target of CHIR signaling, could physically interact with STAT3 and suppress STAT3-dependent gene expression by blocking its DNA-binding activity [37]. Further investigations are needed to resolve this complicated relationship. We further identified *Klf4* as a missing target of CHIR, and demonstrated that CHIR treatment or transient β-catenin expression effectively enhanced *Klf4* activity. We used a small molecule, BIO, as a positive control, which supported the above results that CHIR was able to promote *Klf4* expression. Interestingly, protein expression level of *Klf4* was higher but *Klf4* mRNA expression was lower after CHIR treatment compared with BIO treatment in mESCs (Fig 3B). This phenomenon might be attributable to the very potent inhibition of GSK3 by CHIR in contrast to BIO. We also noted that *Klf4* expression was reduced to a greater extent in 20 μM CHIR-treated F9 cells in contrast to 15 μM CHIR treatment (Fig 4B). It is possible that F9 cell viability could be influenced by a high concentration of CHIR.

*β*-catenin–DNA interactions have identified motifs that match binding sites for *Klf4* by using *in vivo* biotinylation technology in ESCs [34]. Moreover, efforts to delineate the mechanism by which *β*-catenin influenced *Klf4* expression, have identified a potential Tcf/Lef binding site in the *Klf4* promoter fragment. This phenomenon was demonstrated by the promoter reporter and ChIP assays. However, slightly elevated expression of the truncated promoter also occurred after CHIR treatment (Fig 5F). We speculate that other mechanisms that respond to CHIR signaling could regulate *Klf4* expression. This is because two recent studies have suggested that Klf4, functions as a 5-methylcytosine (5mC) reader, binding to specific methylated and/or unmethylated elements in mESCs [38, 39]. Therefore, we cannot disregard the involvement of epigenetic control via DNA methylation mediated by the post-transcriptional and post-translational modifications of Klf4.

Post-transcriptional modifications of stemness factors mediated by sumoylation [40], ubiquitination [41–43], and miRNAs, function in many important processes, such as expression of self-renewal genes in ESCs, cell cycle control of ESCs, alternative splicing and heart development. Thus, we further investigated miRNAs that potentially target the 3′-UTR of *Klf4* and found that miR-7a could inhibit *Klf4* expression. The 3′-UTR of *Klf4* that we used, contains two putative miR-7a binding sites, consistent with previous studies [31, 32]. We also found that CHIR inhibited miR-7a expression (Fig 6E). However, overexpression of *β*-catenin could not repress miR-7a expression, suggesting a *β*-catenin-independent manner between CHIR signaling and *Klf4* regulation mediated by miR-7a. Recent studies showed that miRNAs are initially transcribed as long, capped and polyadenylated primary miRNA transcripts. The RNase III enzymes, Drosha and Dgcr8 process the pri-miRNAs into approximately 72 bp precursor miRNAs, which are further processed by the RNase Dicer to produce the mature miRNAs [44]. Moreover, the inhibition of GSK3 by CHIR reduces Drosha nuclear localization, resulting in the loss of miRNAs [45], similar to Dgcr8 knockout mESCs, which show a global loss of miRNAs [46]. In addition, Dgcr8 knockout mESCs are defective at differentiation, even under stringent differentiation conditions, consistent with the findings that miRNAs are crucial for normal ESC self-renewal and cellular differentiation by tightly controlling ESC self-renewal and differentiation pathways [46–48]. Thus, CHIR inhibits miR-7a expression, likely by inhibiting GSK3. However, further studies are required to demonstrate the role of CHIR and the miRNA profile of ESCs. Together, these data strongly suggest that CHIR treatment enhances *Klf4* expression by transcriptionally activating *Klf4* in a *β*-catenin-dependent manner and by repressing the expression level of miRNAs that target *Klf4* in a *β*-catenin-independent manner.

## Materials and Methods

### Cell culture and transfection

J1 mESCs purchased from the American Type Culture Collection (Manassas, VA, USA) were cultured without feeders on tissue culture plates coated with 0.1% gelatin. The cells were cultured in knockout Dulbecco’s modified Eagle’s medium supplemented with 15% (v/v) knockout serum replacement, 0.1 mM *β*-mercaptoethanol, 1× non-essential amino acids, 2 mM GlutaMax, 50 U/mL penicillin, 50 μg/mL streptomycin (Life Technologies Inc., Grand Island, NY, USA), and 1000 U/mL LIF (ESGRO, Millipore, USA). The culture of murine F9 EC cells (cell bank of Chinese Academy of Sciences, China) and HEK 293FT cells (ATCC, Manassas, VA, USA) were performed as previously described [49, 50]. Transfections were performed by using Lipofectamine 2000 (Life Technologies Inc., Carlsbad, CA) according to the manufacturer’s instructions. All cell culture reagents were purchased from Gibco (Invitrogen, Carlsbad, CA, USA) unless indicated and sterile cell wells were purchased from Nunclon (Roskilde, Denmark).

### CHIR treatment

CHIR99021 (Santa Cruz, CA, USA) was dissolved in dimethyl sulfoxide (DMSO) and added to cell medium at a final concentration of 3 μM for 24 h unless otherwise specified in J1 mESCs. While F9 ECs were treated with CHIR at a final concentration of 3 μM, 5 μM, 10 μM, 15 μM and 20 μM respectively. Cell medium with an equal volume of DMSO was used as a control.

### Reverse transcription PCR and quantitative real-time PCR analysis

Total RNA was extracted from treated cells using Trizol Reagent (Life Technologies) according to the manufacturer’s instructions. Total RNA (1 μg) was reverse- transcribed using a PrimeScript RT reagents kit (TaKaRa, Dalian, China). Messenger RNA expression was determined by real-time PCR with an ABI StepOne Plus PCR system (Applied Biosystems, CA, USA) using SYBR Premix Ex Taq II (TaKaRa). PCR amplification cycles were programmed for 30 s at 95°C, followed by 40 cycles of 95°C for 5 s, 60°C for 30 s. After each cycle, SYBR green fluorescence was monitored, and the melting curve was analyzed to ensure that a single PCR product was obtained. Data were collected after each annealing step. Glyceraldehyde-3-phosphate dehydrogenase (Gapdh) was used as an endogenous control to normalize for the differences in the amount of total RNA in each sample. The following primers were used: *Klf4* forward primer, 5′-GGCGAGTCTGACATGGCTG-3′; *Klf4* reverse primer, 5′-GCTGGACGCAGTGTCTTCTC-3′; *Nanog* forward primer, 5′-CACCCACCCATGCTAGTCTT-3′; *Nanog* reverse primer, 5′-ACCCTCAAACTCCTGGTCCT-3′; *Tfcp2l1* forward primer, 5′-CAGCCCGAACACTACAACCAG-3′; *Tfcp2l1* reverse primer, 5′-*CAGCCGGATTTCATACGACTG*-3′; *Axin2 forward primer*, 5′-*GGGGGAAAACACAGCTTACA*-3′; *Axin2* reverse primer, 5′-TTGACTGGGTCGCTTCTCTT-3′; *Gapdh* forward primer, 5′-GTGTTCCTACCCCCAATGTGT-3′; *Gapdh* reverse primer, 5′-ATTGTCATACCAGGAAATGAGCTT-3′; For miRNA RT-qPCR analysis, the total RNA (2 μg) was reverse transcribed using a miScript II RT kit (Qiagen, China Shanghai Co. Ltd.) with 5 × miScript HiSpec buffer to obtain mature miRNA. Real-time PCR was performed in triplicate for each sample using miScript SYBR Green PCR kit (Qiagen). We used the endogenous reference RNA RNU6B to normalize the amount of template added. The following primers were used: miR-7a primer 5′-GGGGGGGTGGAAGACTAGTGA-3′; U6 Reverse primer 5′-CTCGCTTCGGCAGCACA-3′; Universal miRNA Forward primer 5′-TGAATCGAGCACCAGTTACGCATGCCGAGGTCGACTTCCTAGA-3′; Relative expression of genes was evaluated and expressed as 2^−ΔΔCT^ following previously described procedures.

### Immunofluorescence staining

J1 mESCs were treated with DMSO or 3 μM CHIR for 24 h on gelatin-coated 12-well plates. The medium was discarded, and the cells were washed twice with phosphate-buffered saline before being fixed and permeabilized with immunostaining fix solution (Beyotime, Jiangsu, China) for 10 min. After blocking with immunostaining blocking buffer (Beyotime) for 1 h, the cells were incubated with primary antibody in dilution buffer (Beyotime) overnight at 4°C and then with Alexa Fluor 555-secondary antibody (Beyotime) for 2 h at room temperature in the dark. After each step, the cells were washed thrice with immunolstaining wash buffer for 5 min before the next step. DAPI (4′, 6-diamidino-2-phenylin-dole) staining was performed after secondary antibody incubation for 10 min at room temperature. The primary antibodies and dilutions used were as follows: rabbit anti-Klf4 (Abcam, Cambridge, UK; 1:500), rabbit anti-Klf4 (Boster, Wuhan, China; 1:500), mouse anti-Oct4 (Santa Cruz, CA, USA; 1:500), rabbit anti-Cdx2 (Santa Cruz; 1:500), and rabbit anti-Nanog (Cell Signaling Technology, Danvers, MA; 1:500). All reagents not indicated were purchased from the Beyotime Institute of Biotechnology (Beyotime). Immunofluorescence staining was visualized and imaged by a confocal microscope (Nikon, Tokyo, Japan).

### Gene ontology (GO) analysis

Gene classification and biological functional annotations were performed using the online DAVID database v6.7 [51, 52]. Gene symbols of differentially expressed genes (fold change > 2, P < 0.05) in DMSO and CHIR were submitted to DAVID. The mouse database was used for Gene Ontology annotation and reference. After functional annotation chart analysis using default parameters, the most significant category (FDR < 0.05) within each of the most significant clusters was selected.

### Plasmid constructs

Promoter sequence of *Klf4* (−1124/+240) containing a Tcf/Lef binding site (wild type, WT) was amplified from the genomic DNA of ES cells by PCR. The PCR primers were as follows: forward primer: 5′-GGGGTACCGGGAAAAGGAGTAATAAGACTAAAA-3′ (underlined letters indicate a KpnI restriction site) and reverse primer: 5′-CCCAAGCTTCCGCCAGGTGAGAATGGCCG-3′ (underlined letters indicate a Hind III restriction site). It was then cloned into the luciferase reporter vector pGL4.10 (Promega, Madison, WI), yielding pGL-*Klf4* (−1124/+240) WT. To disrupt the Tcf/Lef-binding motifs, forward primer 5′-GGGGTACCGGGAAAAGGAGTAATAAGACTA**CCAACACG**ACTGC-3′ (underlined letters indicate a KpnI restriction site, and the bold fonts indicate a mutant Tcf/Lef-binding motifs) and reverse primer: 5′-CCCAAGCTTCCGCCAGGTGAGAATGGCCG-3′ (underlined letters indicate a Hind III restriction site) were used to generate pGL-*Klf4* (−1124/+240) Mut. 5′-deletion construct was generated by PCR using the forward primer 5′-GGGGTACCCTCCCGGTAGGCAAGGGCCTGAGGT-3′ (underlined letters indicate a KpnI restriction site) and reverse primer 5′-CCCAAGCTTCCGCCAGGTGAGAATGGCCG-3′ (underlined letters indicate a Hind III restriction site), yielding pGL-*Klf4* (−1081/+240). The full-length coding sequence of β-catenin was amplified from mESC cDNA by PCR. The PCR primers were as follows: forward primer: 5′-CCGCTCGAG**CG**ATGGCTACTCAAGCTGACCTGATG-3′ (underlined letters indicate an XhoI restriction site and the bold fonts were inserted to make sure the correctness of open reading frame) and reverse primer: 5′-ATTTGCGGCCGCTTACAGGTCAGTATCAAACCAGGC-3′ (underlined letters indicate a NotI restriction site). It was then cloned into the pCMV-Myc vector, yielding pCMV-Myc-*β*-catenin. To obtain stabilization expression of the *β*-catenin protein, we constructed the mutation form of *β*-catenin (pCDNA3.1-*β*-catenin S37A). To generate a miR-7a expression vector, a fragment carrying pre-miR-7a was amplified from genomic DNA of J1 ESCs and cloned into the EcoRI and NotI sites of the pCDH-CMV-MCS-EF1-coGFP vector (System Biosciences, Mountain View, CA, USA). Expression vectors of miR-128, miR-152, and miR-363 were constructed using the same method. The PCR primers were as follows: miR-7a forward primer: 5′-CGGAATTCGTCAGTTGACCCTTAGCTTTATGT-3′ (underlined letters indicate a EcoRI restriction site) and reverse primer 5′-ATAAGAATGCGGCCGCACAGCACCATCATTATCTGTCCT-3′ (underlined letters indicate a NotI restriction site); miR-128 forward primer: 5′-CGGAATTCGCAGCTTCTCCTTATGTGCTTAT-3′ (underlined letters indicate a EcoRI restriction site) and reverse primer 5′-ATAAGAATGCGGCCGCGATGTCTAGTAATGAGTTTGGCATG-3′ (underlined letters indicate a NotI restriction site); miR-152 forward primer: 5′-CGGAATTCGAGATTCTGGCGGGACGAGGGAGGA-3′ (underlined letters indicate a EcoRI restriction site) and reverse primer 5′-ATAAGAATGCGGCCGCTCCAGCGGGGAGGGGTTACCTTGGC-3′ (underlined letters indicate a NotI restriction site); miR-363 forward primer: 5′-CGGAATTCTTCAGCATCCATGCCCATTCAT-3′ (underlined letters indicate a EcoRI restriction site) and reverse primer 5′-ATAAGAATGCGGCCGCCACCCTGAGTGAGTTCCAGGACA-3′ (underlined letters indicate a NotI restriction site); The 3’-UTRs of *Klf4* (NM_010637.3) that contain two putative miRNA binding sites [32], were amplified through PCR from J1 ESC cDNA and cloned into multiple cloning sites (MCS) of psiCHECK-2 Vector (Promega). The PCR primers were as follows: forward primer: 5′-CCGCTCGAGATCCCACGTAGTGGATGTGACCCAC -3′ (underlined letters indicate a XhoI restriction site) and reverse primer: 5′-ATAAGAATGCGGCCGCAACTTATTTCTCACCTTGAGTATGC-3′ (underlined letters indicate a NotI restriction site). All plasmids were confirmed by sequencing.

### Western blot analysis

Total protein of J1 mESCs or F9 EC cells was extracted, and its concentration was determined using the bicinchoninic acid protein assay (Beyotime). Equal amounts of protein were resolved by sodium dodecyl sulfate-polyacrylamide gel electrophoresis. Then the proteins were transferred to the polyvinylidene fluoride membranes (Millipore). After blocking for 3 h at room temperature in 10% nonfat dry milk in TBST containing 0.05% Tween-20, the membranes were incubated with primary antibodies overnight at 4°C and then after washes were incubated with HRP-conjugated secondary antibodies for 2 h at room temperature. Peroxidase activity was detected through autography using SuperSignal west pico substrate (Pierce/Thermo Scientific, Rockford, IL, USA). Primary antibodies included rabbit anti-Klf4 (Abcam, Cambridge, UK, 1:1000) and anti-β-catenin (Santa Cruz, CA, USA, 1:1000). Rabbit monoclonal anti-PCNA (Abcam. 1:1000). Rabbit monoclonal anti-GAPDH (Sigma. 1:5000).

### Luciferase assays

For reporter assays, transfection efficiencies were normalized to Renilla plasmid pRL-TK (Promega, Madison, WI) values, which served as an internal control. DNA concentrations were kept constant with an empty expression vector. The cells were harvested after transfection, and luciferase activity was measured using the Dual-Luciferase Reporter (DLR) assay system (Promega) according to the supplier’s recommendations. Briefly, the cells were transfected with reporter constructs, and a Renilla luciferase plasmid was co-transfected as an internal control by Lipofectamine™2000 according to the manufacturer’s instructions. The cells were lysed with 100 μL of passive lysis buffer (Promega) after transfection. The cell lysate was vortexed and then briefly centrifuged. A 20 μL aliquot of the cell lysate was assayed for luciferase activity using VICTORX5 Multilabel Plate Reader (PerkinElmer, Cetus, Norwalk, USA). All transfections were repeated thrice.

### Topflash assays

The pSuperTOPFlash luciferase reporter was constructed by inserting seven copies of the TCF/LEF binding site (AGATCAAAGG) into the pTA-luc vector, while pFOPFlash was previously constructed using second amplification from M51 Super 8x FOPFlash vector (Addgene plasmid # 12457) and cloned into the XhoI and BglII sites of the pTA-luc vector (Clontech, Mountain View, CA). These constructs contain a firefly luciferase reporter under the control of seven repeats the wild-type or mutant Tcf binding site upstream of minimal TA promoter. pFopflash containing mutated Tcf binding site was used as a negative control. The pRL-TK luciferase reporter construct was used as an internal standard of transfection. Transfection of plasmid DNA and assay for luciferase activity were performed as described previously.

### RNA Interference

For *β*-catenin knockdown in J1 ES cells, short interfering RNAs (siRNAs) that target mouse β-catenin and negative control siRNA-NC were purchased from Shanghai GenePharma (Shanghai, China). siRNA sequences were: si-*β*-catenin: 5′-GCCUUAGUAAACAUAAUGA-3′. For RNA interference experiments, J1 mESCs were transfected with the indicated siRNAs (50 nM final concentrations) using Lipofectamine 2000.

### Chromatin immunoprecipitation assay

The protocol for chromatin immunoprecipitation assays (ChIP) has been described previously [49]. Briefly, J1 mESCs were treated with 3 μM CHIR for 24 h on gelatin-coated plates. Cells were cross-linked for 10 minutes at room temperature with 1% (wt/vol) formaldehyde and the reaction subsequently quenched with 125 mM glycine. Genomic DNA was isolated and sheared to average lengths of 300–500 base pair (bp) by micrococcal nuclease according to the manufacturer’s protocol (Pierce). Rabbit anti-*β*-catenin (Santa Cruz, CA, USA) and rabbit anti-IgG (pierce) were used for immunoprecipitation. ChIP enrichment was performed by qPCR. Fold-enrichment was determined by normalizing threshold cycle values of *β*-catenin ChIP against IgG ChIP. The following target primers were used for ChIP-qPCR: *Klf4* forward primer, 5′-ACTGAGGGTAGTGGGGAATGG-3′ *Klf4* reverse primer 5′-GTGATCCTGCGCTGGGAAGAG-3′.

### Statistical analysis

The data in graphs are expressed as the mean ± SD. The difference between two groups was compared by a two-tailed paired Student’s t-test, and significance was set at P-values < 0.05. The difference between three or more groups was compared by an analysis of variance (ANOVA), and significance of differences was determined by post hoc testing.

## Supporting Information

S1 Fig CHIR promotes *Klf4* expression in the absence of LIF. **(A, B)**: J1 mESCs were treated with 3 μM CHIR or equal volume of DMSO in the presence or absence of LIF for 24 h, RT-qPCR was used to detect the expression of *Klf4* and *Map2*. Data are presented as the mean ± SD of three independent experiments. (*p < 0.05; **p < 0.01).
(TIF)

S2 Fig Structure of TCF-responsive TOPFlash reporter construct. This construct contain a firefly luciferase reporter under the control of seven repeats the wild-type Tcf binding site upstream of minimal TA promoter (pTA). Abbreviation luc represent firefly luciferase gene.
(TIF)

S3 Fig The expression of cytosolic *β*-catenin. **(A, B)**: CHIR treatment or *β*-catenin overexpression promotes cytosolic *β*-catenin expression. J1 mESCs were treated with 3 μM CHIR or equal volume of DMSO (A), or transfected with pCDNA3.1-*β*-catenin s37a / pCDNA3.1 control plasmid (B) for 48 h, Cell cytosolic lysates were extracted and the expression of *β*-catenin was analyzed by western blot. Relative expression levels were compared with Gapdh.
(TIF)

S4 Fig The expression level of miR-7a. MiR-7a expression vector pCDH-mir-7a and their negative control pCDH-GFP were transfected into J1 mESCs, and miR-7a expression was detected by RT-qPCR. U6 was used to normalize template levels. Data are presented as the mean ± SD of three independent experiments (**p < 0.01).
(TIF)

S1 Table Differentially expressed transcripts in CHIR99021 treated J1 mESCs. Fold change (FC) values are provided in comparison with the control J1 mESCs which were maintained in standard ESC medium without the addition of CHIR99021. (FC < 0.2, p < 0.05).
(DOC)

S2 Table miRNAs that potentially target Klf4 were predicted using multiple databases. Targetscan, Pic tar, Microsom Targets and DTANA-lab microT 3.0 were used to predict the potential miRNAs that may target Klf4 in mouse.
(DOCX)

S3 Table The scores of the different miRNAs that target *Klf4* in the Targetscan. (DOCX)

S4 Table The scores of the different miRNAs that target *Klf4* in the PicTar. (DOCX)
