# Supplementary figures and images for: CHIR99021 enhances Klf4 Expression through β-Catenin Signaling and miR-7a Regulation in J1 Mouse Embryonic Stem Cells
Source: PLoS One. 2016 Mar 3;11(3):e0150936. doi: 10.1371/journal.pone.0150936 (PMC4777400; doi:10.1371/journal.pone.0150936)

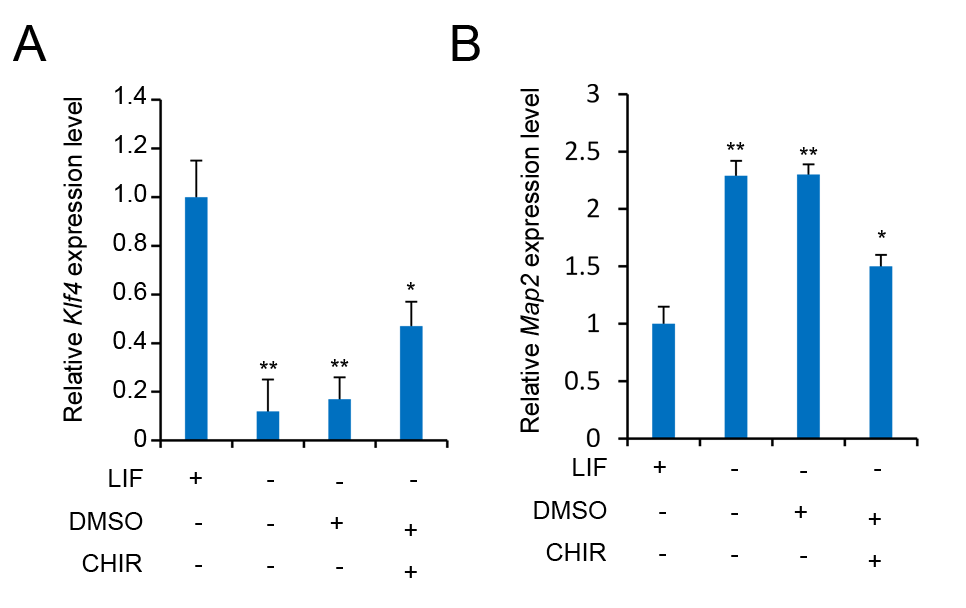

Supplement: S1 Fig — (A, B): J1 mESCs were treated with 3 μM CHIR or equal volume of DMSO in the presence or absence of LIF for 24 h, RT-qPCR was used to detect the expression of Klf4 and Map2. Data are presented as the mean ± SD of three independent experiments. (*p < 0.05; **p < 0.01). (TIF) [file pone.0150936.s001.tif]

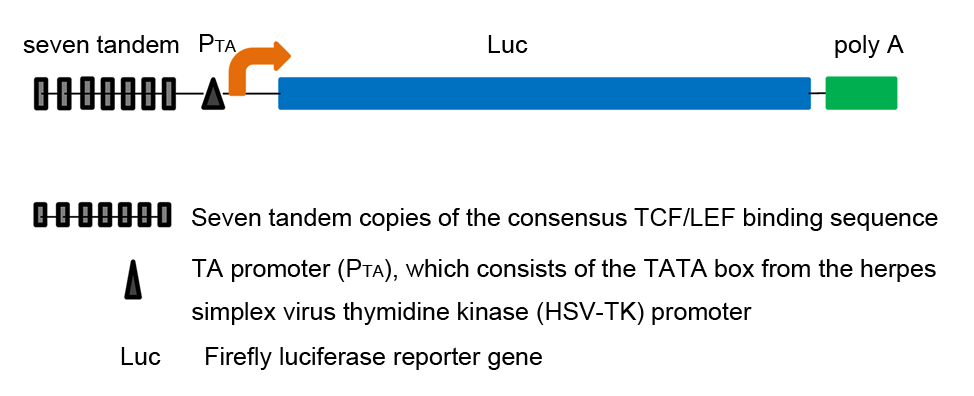

Supplement: S2 Fig — This construct contain a firefly luciferase reporter under the control of seven repeats the wild-type Tcf binding site upstream of minimal TA promoter (pTA). Abbreviation luc represent firefly luciferase gene. (TIF) [file pone.0150936.s002.tif]

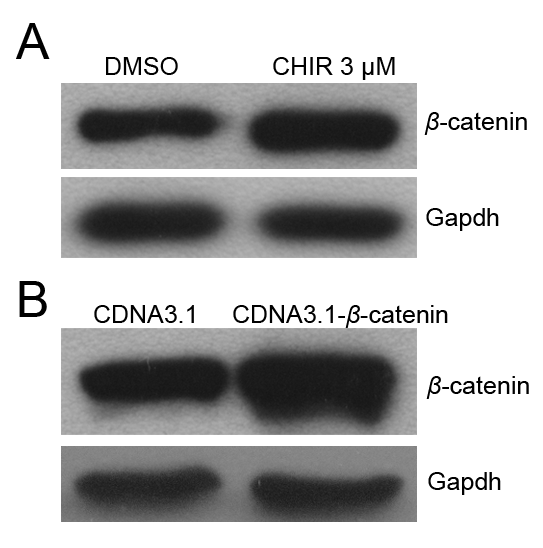

Supplement: S3 Fig — (A, B): CHIR treatment or β-catenin overexpression promotes cytosolic β-catenin expression. J1 mESCs were treated with 3 μM CHIR or equal volume of DMSO (A), or transfected with pCDNA3.1-β-catenin s37a / pCDNA3.1 control plasmid (B) for 48 h, Cell cytosolic lysates were extracted and the expression of β-catenin was analyzed by western blot. Relative expression levels were compared with Gapdh. (TIF) [file pone.0150936.s003.tif]

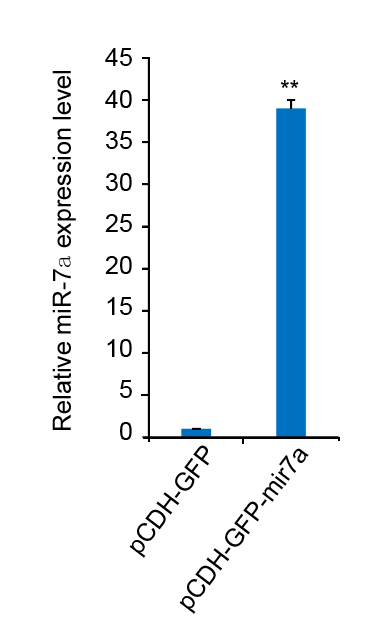

Supplement: S4 Fig — MiR-7a expression vector pCDH-mir-7a and their negative control pCDH-GFP were transfected into J1 mESCs, and miR-7a expression was detected by RT-qPCR. U6 was used to normalize template levels. Data are presented as the mean ± SD of three independent experiments (**p < 0.01). (TIF) [file pone.0150936.s004.tif]
