# Supplementary material for: CHIR99021 enhances Klf4 Expression through β-Catenin Signaling and miR-7a Regulation in J1 Mouse Embryonic Stem Cells
Source: PLoS One. 2016 Mar 3;11(3):e0150936. doi: 10.1371/journal.pone.0150936 (PMC4777400; doi:10.1371/journal.pone.0150936)
Supplement: S1 Table — Fold change (FC) values are provided in comparison with the control J1 mESCs which were maintained in standard ESC medium without the addition of CHIR99021. (FC < 0.2, p < 0.05). (DOC) [file pone.0150936.s005.doc]

**Table S1. Differentially expressed transcripts in CHIR99021 treated J1 ES cells.** Fold change (FC) values are provided in comparison with the control ESCs which were maintained in standard ES cells medium without the addition of CHIR99021. (FC < 0.2, p < 0.05).

| **ProbeName** | **GeneSymbol** | **p CHIR VS J1** | **FC CHIR VS J1** |
| --- | --- | --- | --- |
| **A_55_P2029161** | **T** | **0.000943628** | **267.8247418** |
| **A_51_P404193** | **Sp5** | **2.65955E-05** | **213.5309177** |
| **A_52_P177490** | **Cdx2** | **0.000202775** | **134.7972512** |
| **A_55_P2157205** | **Cdx1** | **2.52579E-06** | **83.16223733** |
| **A_55_P2081488** | **Pglyrp1** | **8.31802E-06** | **54.74690998** |
| **A_55_P2164659** | **Tlx2** | **9.67095E-06** | **50.1974927** |
| **A_52_P144310** | **Gad1** | **3.7413E-06** | **32.75568847** |
| **A_55_P2000783** | **Axin2** | **1.38956E-07** | **30.68868876** |
| **A_52_P94521** | **2510009E07Rik** | **0.017045128** | **17.45852373** |
| **A_52_P128134** | **Foxd1** | **0.000162527** | **17.23552214** |
| **A_55_P2002287** | **Msx3** | **0.001272674** | **17.20824343** |
| **A_52_P532456** | **Plagl1** | **1.39871E-05** | **16.50426897** |
| **A_55_P1961968** | **Plagl1** | **0.00092672** | **15.25868785** |
| **A_52_P657360** | **Tnni1** | **0.004447534** | **14.0915633** |
| **A_55_P2394308** | **Fst** | **4.97562E-05** | **14.0006131** |
| **A_66_P134405** | **Axin2** | **1.15965E-06** | **13.15119203** |
| **A_51_P145220** | **Nefm** | **2.07465E-05** | **12.7657763** |
| **A_52_P56751** | **Lcp1** | **0.00263089** | **12.6339045** |
| **A_51_P147123** | **Ntn1** | **0.000949656** | **12.50074264** |
| **A_55_P2152771** | **Lhfpl2** | **0.000226728** | **12.35284948** |
| **A_55_P2024948** | **Hoxb8** | **0.001205493** | **11.63546127** |
| **A_55_P2162935** | **Ntn1** | **1.08713E-06** | **11.14689917** |
| **A_51_P116932** | **Lad1** | **2.45481E-06** | **11.1258668** |
| **A_55_P2028832** | **Bex6** | **0.006452101** | **10.88480859** |
| **A_55_P2014932** | **Gm2245** | **8.10591E-05** | **10.87221033** |
| **A_55_P2097508** | **Mcc** | **0.000598673** | **10.19581832** |
| **A_55_P2105180** | **Bhmt** | **0.005594462** | **10.16925134** |
| **A_55_P2068892** | **Il6ra** | **1.79228E-06** | **9.195360696** |
| **A_51_P245631** | **Rftn2** | **0.000990195** | **8.816544138** |
| **A_55_P1997569** | **Nkx6-2** | **0.000207716** | **8.759110078** |
| **A_52_P93910** | **Nrp2** | **1.81927E-06** | **8.521865411** |
| **A_51_P518600** | **Atp6v1c2** | **0.000310418** | **8.473971833** |
| **A_55_P2080592** | **Gm4934** | **1.13978E-05** | **8.340148856** |
| **A_51_P470304** | **Nkx1-2** | **0.006626306** | **8.001485506** |
| **A_55_P2074688** | **Hoxc4** | **0.000375243** | **7.915110364** |
| **A_51_P375783** | **Prap1** | **7.21665E-07** | **7.690146417** |
| **A_55_P2232023** | **AI448005** | **0.000402451** | **7.621263871** |
| **A_51_P411345** | **Mogat2** | **3.922E-07** | **7.414213536** |
| **A_51_P499838** | **Bst1** | **0.00147299** | **7.38496133** |
| **A_51_P394115** | **Aadac** | **0.006253756** | **7.351700021** |
| **A_55_P1999833** | **Odz1** | **6.77821E-05** | **7.307266715** |
| **A_55_P1979833** | **Cited1** | **1.55014E-05** | **6.919802362** |
| **A_52_P338066** | **Ubd** | **4.82402E-05** | **6.736634062** |
| **A_55_P2060592** | **Hoxa1** | **0.000107235** | **6.533801686** |
| **A_55_P2079619** | **Rnf43** | **1.26444E-05** | **6.390050798** |
| **A_55_P2105181** | **Bhmt** | **3.84376E-06** | **6.224973231** |
| **A_55_P1953169** | **Saa3** | **2.87856E-06** | **6.158063739** |
| **A_55_P1984976** | **Wnt5b** | **1.56232E-06** | **6.155308505** |
| **A_65_P16059** | **Tgfbr3** | **3.75407E-05** | **6.044005612** |
| **A_55_P2113160** | **Mal** | **0.000116591** | **6.013721818** |
| **A_52_P195839** | **Ctsc** | **0.000178993** | **6.002620804** |
| **A_52_P500274** | **Ntrk3** | **9.64185E-05** | **5.842387167** |
| **A_55_P2004208** | **Defa-rs2** | **4.39665E-05** | **5.83051923** |
| **A_66_P135651** | **Capn9** | **1.6521E-05** | **5.757772953** |
| **A_52_P175376** | **Tcfcp2l1** | **4.53653E-05** | **5.727594407** |
| **A_55_P2006808** | **Ntrk3** | **0.000154205** | **5.713187718** |
| **A_55_P2365710** | **B130046B21Rik** | **2.82367E-05** | **5.673016264** |
| **A_51_P287198** | **Krt23** | **0.000429086** | **5.63057659** |
| **A_52_P275069** | **Gm6792** | **1.58968E-05** | **5.590426556** |
| **A_55_P2049976** | **Gm2903** | **3.2797E-05** | **5.524071144** |
| **A_52_P16752** | **Aox3** | **0.001213004** | **5.500927034** |
| **A_51_P347529** | **Nek5** | **0.001521602** | **5.48265629** |
| **A_51_P279100** | **Ptgs1** | **0.000390509** | **5.462550328** |
| **A_55_P2153633** | **Snrpc** | **0.003230842** | **5.44804631** |
| **A_55_P2044385** | **Fgfbp3** | **0.000101212** | **5.374997188** |
| **A_55_P2359797** | **Nod2** | **7.63832E-05** | **5.371105155** |
| **A_52_P468343** | **Bdh1** | **1.20755E-05** | **5.343298693** |
| **A_55_P2173457** | **1700097N02Rik** | **0.000161312** | **5.210937074** |
| **A_55_P2319035** | **AW011956** | **1.31025E-05** | **5.184319866** |
| **A_55_P2055844** | **Fbxo25** | **0.014065328** | **5.172874415** |
| **A_51_P386899** | **Mfsd7c** | **1.02908E-05** | **5.171105242** |
| **A_52_P487436** | **Nags** | **0.000174348** | **5.081233045** |
| **A_55_P2054062** | **Chst2** | **8.58261E-06** | **5.075766257** |
| **A_52_P203560** | **Fzd10** | **2.61529E-07** | **5.037758626** |
| **A_55_P1999790** | **Pkdcc** | **2.14365E-05** | **0.19469661** |
| **A_51_P241995** | **Col5a3** | **0.000640539** | **0.192849503** |
| **A_55_P1981836** | **BC020535** | **0.000186062** | **0.191119969** |
| **A_51_P259029** | **Dusp26** | **0.000338768** | **0.19031947** |
| **A_55_P1958275** | **Bcl11a** | **5.0998E-07** | **0.185885688** |
| **A_55_P1966064** | **Gm1564** | **0.003099149** | **0.185494129** |
| **A_55_P2184123** | **Atp1a4** | **0.013873428** | **0.185327558** |
| **A_51_P315904** | **Gadd45g** | **1.19246E-07** | **0.182238961** |
| **A_52_P586928** | **Pdyn** | **9.15263E-05** | **0.181081567** |
| **A_55_P2153391** | **Palm2** | **0.002309025** | **0.177976179** |
| **A_51_P227275** | **Csn3** | **0.009591387** | **0.176356408** |
| **A_55_P2132781** | **Slc16a2** | **0.00763123** | **0.172502375** |
| **A_55_P2100586** | **Cass4** | **0.005101041** | **0.172351186** |
| **A_51_P367866** | **Egr1** | **2.25593E-07** | **0.172167014** |
| **A_51_P339540** | **Cdkn1c** | **9.81656E-06** | **0.169158198** |
| **A_51_P246066** | **Slamf9** | **2.35609E-06** | **0.162557223** |
| **A_55_P2046448** | **Gm12581** | **8.07014E-05** | **0.162377022** |
| **A_52_P165773** | **Prmt8** | **3.01094E-05** | **0.158595646** |
| **A_52_P656699** | **Actn3** | **4.71662E-07** | **0.145183224** |
| **A_52_P253044** | **Syt13** | **0.000408859** | **0.138749114** |
| **A_52_P285470** | **Lrp2** | **5.33052E-05** | **0.134511072** |
| **A_51_P203955** | **Gbp2** | **0.001008348** | **0.133377304** |
| **A_55_P1964348** | **Gdpd2** | **0.019016152** | **0.132729277** |
| **A_66_P125035** | **Gm10394** | **3.61916E-06** | **0.124016722** |
| **A_55_P2047188** | **Fgf1** | **4.64227E-05** | **0.122254141** |
| **A_55_P2132873** | **Gdap1l1** | **0.000323815** | **0.12132132** |
| **A_51_P354706** | **Lefty1** | **1.36108E-05** | **0.119993057** |
| **A_55_P2009787** | **Atp1a4** | **0.001508127** | **0.119756837** |
| **A_52_P340073** | **Efnb2** | **7.68585E-07** | **0.112036332** |
| **A_52_P379277** | **Enpp3** | **2.6767E-05** | **0.109836363** |
| **A_51_P345367** | **Psmb8** | **0.001035929** | **0.109424677** |
| **A_55_P2087182** | **Car4** | **0.001613331** | **0.106466469** |
| **A_55_P1973159** | **Serpinf1** | **0.000232213** | **0.106119393** |
| **A_51_P306017** | **Dll1** | **8.35662E-07** | **0.098419998** |
| **A_55_P1953143** | **Wt1** | **3.34298E-06** | **0.082821672** |
| **A_51_P461040** | **Crct1** | **0.000868066** | **0.067305647** |
| **A_66_P112596** | **Gm9564** | **1.75924E-05** | **0.064493638** |
| **A_55_P2369534** | **C430049E01Rik** | **0.003004394** | **0.057889453** |
| **A_55_P1978571** | **Otx2** | **9.07929E-05** | **0.029091602** |
