# Supplementary material for: CHIR99021 enhances Klf4 Expression through β-Catenin Signaling and miR-7a Regulation in J1 Mouse Embryonic Stem Cells
Source: PLoS One. 2016 Mar 3;11(3):e0150936. doi: 10.1371/journal.pone.0150936 (PMC4777400; doi:10.1371/journal.pone.0150936)
Supplement: S2 Table — Targetscan, Pic tar, Microsom Targets and DTANA-lab microT 3.0 were used to predict the potential miRNAs that may target Klf4 in mouse. (DOCX) [file pone.0150936.s006.docx]

**Table S2.** **miRNAs that potentially target Klf4 were predicted using multiple databases.**

Targetscan, Pic tar, Microsom Targets and DIANA-lab microT 3.0 were used to predict the potential miRNAs that may target Klf4 in mouse.

| **Targetscan** | **Pic tar** | **Microcosm Targets** | **DIANA-lab microT 3.0** |
| --- | --- | --- | --- |
| [mmu-miR-200c](http://www.mirbase.org/cgi-bin/mirna_entry.pl?acc=mmu-miR-200c) | [mmu-miR-32](http://www.mirbase.org/cgi-bin/mirna_entry.pl?acc=mmu-miR-200c) | [mmu-miR-148b](http://www.mirbase.org/cgi-bin/mirna_entry.pl?acc=mmu-miR-200c) | [mmu-miR-32](http://www.mirbase.org/cgi-bin/mirna_entry.pl?acc=mmu-miR-200c) |
| [mmu-miR-200b](http://www.mirbase.org/cgi-bin/mirna_entry.pl?acc=mmu-miR-200b) | [mmu-miR-7b](http://www.mirbase.org/cgi-bin/mirna_entry.pl?acc=mmu-miR-200b) | [mmu-miR-19a](http://www.mirbase.org/cgi-bin/mirna_entry.pl?acc=mmu-miR-200b) | [mmu-miR-92](http://www.mirbase.org/cgi-bin/mirna_entry.pl?acc=mmu-miR-200b) |
| [mmu-miR-429](http://www.mirbase.org/cgi-bin/mirna_entry.pl?acc=mmu-miR-429) | [mmu-miR-449](http://www.mirbase.org/cgi-bin/mirna_entry.pl?acc=mmu-miR-429) | [mmu-miR-19b](http://www.mirbase.org/cgi-bin/mirna_entry.pl?acc=mmu-miR-429) | [mmu-miR-29a](http://www.mirbase.org/cgi-bin/mirna_entry.pl?acc=mmu-miR-429) |
| [mmu-miR-26a](http://www.mirbase.org/cgi-bin/mirna_entry.pl?acc=mmu-miR-429) | [mmu-miR-7](http://www.mirbase.org/cgi-bin/mirna_entry.pl?acc=mmu-miR-429) | [mmu-miR-32](http://www.mirbase.org/cgi-bin/mirna_entry.pl?acc=mmu-miR-429) | [mmu-miR-29b](http://www.mirbase.org/cgi-bin/mirna_entry.pl?acc=mmu-miR-429) |
| [mmu-miR-26b](http://www.mirbase.org/cgi-bin/mirna_entry.pl?acc=mmu-miR-429) | [mmu-miR-124a](http://www.mirbase.org/cgi-bin/mirna_entry.pl?acc=mmu-miR-429) | [mmu-miR-466f-5p](http://www.mirbase.org/cgi-bin/mirna_entry.pl?acc=mmu-miR-429) | [mmu-miR-29c](http://www.mirbase.org/cgi-bin/mirna_entry.pl?acc=mmu-miR-429) |
| mmu-miR-1395 | mmu-miR-92 | mmu-miR-140 | mmu-miR-363 |
| mmu-miR-145 | mmu-miR-547 | mmu-miR-669b | mmu-miR-449 |
| mmu-miR-125 | mmu-miR-31 | mmu-miR-219 | mmu-miR-34a |
| mmu-miR-29a | mmu-miR-315b | mmu-miR-29b | mmu-miR-148b |
| mmu-miR-29b | mmu-miR-29b | mmu-miR-29a | mmu-miR-152 |
| mmu-miR-29c | mmu-miR-29a | mmu-miR-367 | mmu-miR-137 |
| mmu-miR-146 | mmu-miR-29c | mmu-miR-199a-5p | mmu-miR-381 |
| mmu-miR-107 | mmu-miR-34a | mmu-miR-494 | mmu-miR-367 |
| mmu-miR-103 | mmu-miR-34c | mmu-miR-7a | mmu-miR-135b |
| mmu-miR-7a | mmu-miR-130a | mmu-miR-136 | mmu-miR-135a |
| mmu-miR-7b | mmu-miR-130b | mmu-miR-382 | mmu-miR-25 |
| mmu-miR-148 | mmu-miR-148a | mmu-miR-154 | mmu-miR-467a |
| mmu-miR-152 | mmu-miR-148b | mmu-miR-128a | mmu-miR-467b |
| mmu-miR-128 | mmu-miR-152 | mmu-miR-547 | mmu-miR-721 |
| mmu-miR-137 | mmu-miR-301 | mmu-miR-381 | mmu-miR-301 |
| mmu-miR-25 | mmu-miR-128a | mmu-miR-376a | mmu-miR-130a |
| mmu-miR-32 | mmu-miR-128b | mmu-miR-687 | mmu-miR-124a |
| mmu-miR-92a | mmu-miR-346 | mmu-miR-92a | mmu-miR-200c |
| mmu-miR-92b | mmu-miR-139 | mmu-miR-92b | mmu-miR-200b |
| mmu-miR-363 | mmu-miR-1 | mmu-miR-363 | mmu-miR-26a |
| mmu-miR-367 | mmu-miR-206 | mmu-miR-409-3p | mmu-miR-26b |
| mmu-miR-375 | mmu-miR-10a | mmu-miR-219 | mmu-miR-429 |
| mmu-miR-346 | mmu-miR-363 |  |  |
| mmu-miR-300 | mmu-miR-25 |  |  |
|  | [mmu-miR-367](http://www.mirbase.org/cgi-bin/mirna_entry.pl?acc=mmu-miR-200c) |  |  |
|  | [mmu-miR-135a](http://www.mirbase.org/cgi-bin/mirna_entry.pl?acc=mmu-miR-200b) |  |  |
|  | [mmu-miR-137](http://www.mirbase.org/cgi-bin/mirna_entry.pl?acc=mmu-miR-429) |  |  |
|  | [mmu-miR-200b](http://www.mirbase.org/cgi-bin/mirna_entry.pl?acc=mmu-miR-429) |  |  |
|  | [mmu-miR-200c](http://www.mirbase.org/cgi-bin/mirna_entry.pl?acc=mmu-miR-429) |  |  |
|  | mmu-miR-429 |  |  |
|  | mmu-miR-26a |  |  |
|  | mmu-miR-26b |  |  |
|  | mmu-miR-381 |  |  |
