# Supplementary material for: CHIR99021 enhances Klf4 Expression through β-Catenin Signaling and miR-7a Regulation in J1 Mouse Embryonic Stem Cells
Source: PLoS One. 2016 Mar 3;11(3):e0150936. doi: 10.1371/journal.pone.0150936 (PMC4777400; doi:10.1371/journal.pone.0150936)
Supplement: S3 Table — (DOCX) [file pone.0150936.s007.docx]

**Table S3. The scores of the different miRNAs that target *Klf4* in the Targetscan.**

| **miRNA** | **Seed math** | **Position of Klf4 3' UTR** | **P_CT_** |
| --- | --- | --- | --- |
| [mmu-miR-152](http://www.mirbase.org/cgi-bin/mirna_entry.pl?acc=mmu-miR-152) | 7mer-m8 | 732-738 | 0.32 |
| [mmu-miR-7a](http://www.mirbase.org/cgi-bin/mirna_entry.pl?acc=mmu-miR-152) | 7mer-m8 | 73-79 | 0.64 |
| [mmu-miR-363](http://www.mirbase.org/cgi-bin/mirna_entry.pl?acc=mmu-miR-363) | 8mer | 432-438 | 0.81 |
| [mmu-miR-128](http://www.mirbase.org/cgi-bin/mirna_entry.pl?acc=mmu-miR-363) | 7mer-m8 | 734-740 | 0.36 |

P_CT_, probability of conserved targeting
