# Supplementary material for: CHIR99021 enhances Klf4 Expression through β-Catenin Signaling and miR-7a Regulation in J1 Mouse Embryonic Stem Cells
Source: PLoS One. 2016 Mar 3;11(3):e0150936. doi: 10.1371/journal.pone.0150936 (PMC4777400; doi:10.1371/journal.pone.0150936)
Supplement: S4 Table — (DOCX) [file pone.0150936.s008.docx]

**Table S4. The scores of the different miRNAs that target *Klf4* in the PicTar.**

| **miRNA** | **PicTar score in mouse** | **probabilities** |
| --- | --- | --- |
| mmu-miR-7 | 7.8629 | 0.98 |
| mmu-miR-128a | 2.9254 | 0.95 |
| mmu-miR-152 | 2.9526 | 0.95 |
| mmu-miR-363 | 2.4962 | 0.92 |

**.**
